# The complete chloroplast genome sequence of the *Citrullus colocynthis* L. (Cucurbitaceae)

**DOI:** 10.1080/23802359.2017.1361351

**Published:** 2017-07-31

**Authors:** Qianglong Zhu, Muyue Zhang, Haonan Cui, Chao Fan, Peng Gao, Xuezheng Wang, Feishi Luan

**Affiliations:** a College of Horticulture, Northeast Agricultural University, Harbin, Heilongjiang, China;; b Ministry of Agriculture, Key Laboratory of Biology and Genetic Improvement of Horticulture Crops (Northeast Region), Harbin, Heilongjiang, China;; c Hainan Base of Heilongjiang Agriculture Academy, Sanya, Hainan, China

**Keywords:** *Citrullus colocynthis* L., chloroplast genome, bitter apple, desert watermelon

## Abstract

*Citrullus colocynthis* L. is one of the worldwide famous traditionally medicinal plants and widely applied in watermelon breeding for its multiple resistances. The complete nucleotide sequence of desert watermelon (*Citrullus colocythis* L.) chloroplast genome has been determined in this study. The genome was composed of 157,147 bp containing a pair of inverted repeats (IRs) of 26,149 bp, which was separated by a large single-copy region of 86,851 bp and a small single-copy region of 17,998 bp. A total of 123 genes were predicted including 86 protein-coding genes, eight rRNA genes and 29 tRNA genes. Phylogenetic analysis revealed that *C. colocynthis* were closely related to other two species in the genus *Citrullus*. The complete chloroplast genome of *C. colocynthis* would provide some significant information for Cucurbitaceae evolutionary and genomic studies.

*Citrullus colocynthis* L., commonly known as ‘bitter apple’, is one of the worldwide famous traditionally medicinal plants belonging to the family Cucurbitaceae. Its fruit, leaf and root are well-known for containing large amounts of bioactive components with great medicinal value (da Silva and Hussain 2017). Studies have reported that the extracts of *C. colocynthis* are widely useful in antidiabetic (Huseini et al. 2009), anticancer (Ayyad et al. 2012), antioxidant (Vashishta 2008), analgesic and anti-inflammatory (Marzouk et al. 2010). *C. colocynthis* have been reported to have high resistance against powdery mildew (Davis et al. 2007), and broad mites (Kousik et al. 2007), and drought stress (Si et al. 2009). Therefore, its chloroplast genome was commonly served as potential sources for use in breeding programs aimed at enhancing stress tolerance, pest or disease resistance in cultivated watermelon (Levi and Thomas 2005; Levi et al. 2006; Levi et al. 2011), and was also important for plant taxonomy and phylogenetic system researches (Dane and Lang 2004; Dane et al. 2004; Dane et al. 2007) in watermelon. In this study, we characterized the complete chloroplast genome sequence of *C. colocynthis* to contribute to further pharmacological, breeding, and phylogenetic studies of this plant.

Sample of *C. colocythis* (accession number: PI 374216) was stored in the College of Horticulture of Northeast Agricultural University (126°43′16.7′′E, 45°44′23.8′′N), Harbin, China. Genomic DNA was extracted from fresh leaves and subjected to construct a genomic library and pair-end (2 × 150 bp) sequenced by HiSeq X Ten (BGI, Shenzhen, China). Whole genome sequence data of 4 Gb were generates and trimmed, high quality pair-end reads of 0.4 Gb were randomly extracted using Seqtk, and assembled with using the Plasmidspades.py in SPAdes (v3.10.1) (Bankevich et al. 2012). Contigs representing the chloroplast genome were retrieved, ordered and joined into a single draft sequence by comparison with the chloroplast genome of *Citrullus lanatus* L.subsp *vugaris* (GenBank accession no. NC_032008.1) as a reference (Zhu et al. 2016). The gaps in the chloroplast single draft sequence of *C. colocynthis* were closed by using GapCloser (v1.12-r6). The draft sequence was then confirmed and manually corrected by PE read mapping. Finally, the draft sequence was annotated using an integrated web server, CpGAVAS (Chang et al. 2012), and manually corrected by visual inspection using IGV (Robinson et al. 2011).

The complete chloroplast genome of *C. colocynthis* (GenBank accession number MF357889) is double-stranded circular DNA with 157,147 bp in length with 37.14% GC contents. It shows a typical quadripartite structure containing a small single-copy (LSC) region of 17,998 bp, a large single-copy (SSC) region of 86,851 bp, separated by a pair of inverted repeat regions (IRa and IRb) of 26,149 bp. The chloroplast genome contains 123 genes were predicted, including 86 protein-coding genes, 29 tRNA genes and eight rRNA genes, of which 15 genes were duplicated in IR regions.

The phylogenetic relationship of *C. colocynthis* was deduced by its comparison with other 15 chloroplast genomes in Cucurbitaceae or others based on 56 common protein-coding genes. Phylogenetic tree was built by using a maximum likelihood (ML) method of MEGA (v7.0) with 1000 bootstrap replicates (Tamura et al. 2013). As expected, *C. colocynthis* was closely related to *C. lanatus* ssp. *vulgaris* and *C. lanatus* ssp. *mucosospermus*, forming a clade included in *Citrullus genus*. The *Citrullus* genus and other species in the family Cucurbitaceae were closely clustered into a clade, and other species in different family belonging to same family were also well clustered into their corresponding clades with high bootstrap value (Figure 1).

**Figure 1.**
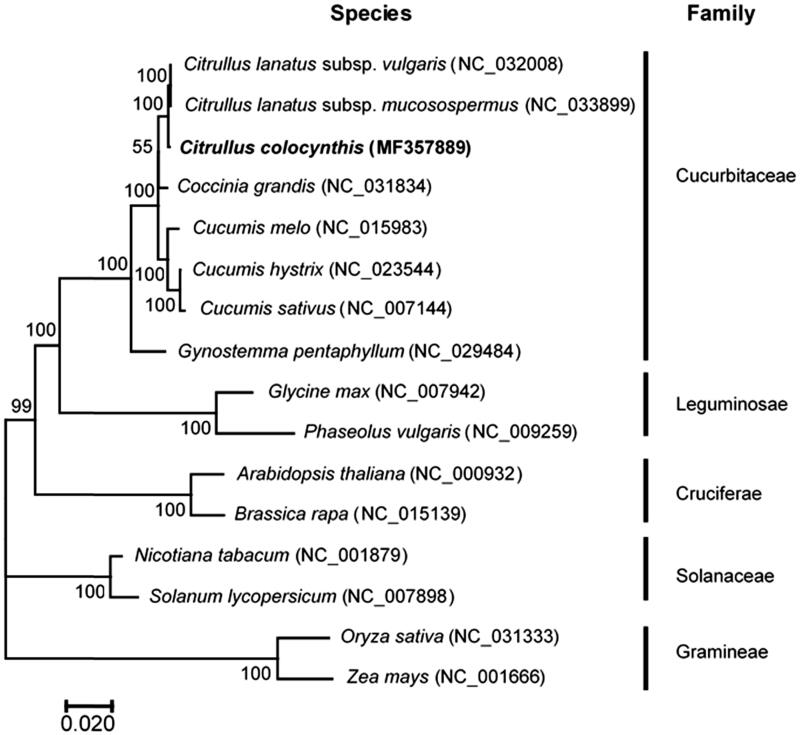
Phylogenetic tree showing relationship between *C. colocynthis* L. and other 15 species belonging to different families. Phylogenetic tree was constructed based on 56 protein-coding genes of chloroplast genomes using maximum likelihood (ML) with 1000 bootstrap replicates. Numbers in each the node indicated the bootstrap support values.

## References

[CIT0001] Ayyad S-EN, Abdel-Lateff A, Alarif WM, Patacchioli FR, Badria FA, Ezmirly ST. 2012 *In vitro* and *in vivo* study of cucurbitacins-type triterpene glucoside from *Citrullus colocynthis* growing in Saudi Arabia against hepatocellular carcinoma. Environ Toxicol Phar. 33:245–251. 10.1016/j.etap.2011.12.010 22245841

[CIT0002] Bankevich A, Nurk S, Antipov D, Gurevich AA, Dvorkin M, Kulikov AS, Lesin VM, Nikolenko SI, Pham S, Prjibelski AD, et al 2012 SPAdes: a new genome assembly algorithm and its applications to single-cell sequencing. J Comput Biol. 19:455–477. 22506599 10.1089/cmb.2012.0021 PMC3342519

[CIT0003] Chang L, Shi L, Zhu Y, Chen H, Zhang J, Lin X, Guan X. 2012 CpGAVAS, an integrated web server for the annotation, visualization, analysis, and GenBank submission of completely sequenced chloroplast genome sequences. BMC Genomics. 13:1–7. 23256920 10.1186/1471-2164-13-715 PMC3543216

[CIT0004] da Silva JAT, Hussain AI. 2017 *Citrullus colocynthis* (L.) Schrad. (colocynth): Biotechnological perspectives. Emir J Food Agr. 29:83–90.

[CIT0005] Dane F, Lang P, Bakhtiyarova R. 2004 Comparative analysis of chloroplast DNA variability in wild and cultivated *Citrullus* species. Theor Appl Genet. 108:958–966. 14634729 10.1007/s00122-003-1512-9

[CIT0006] Dane F, Lang P. 2004 Sequence variation at cpDNA regions of watermelon and related wild species: implications for the evolution of *Citrullus* haplotypes. Am J Bot. 91:1922–1929. 21652338 10.3732/ajb.91.11.1922

[CIT0007] Dane F, Liu J, Zhang C. 2007 Phylogeography of the bitter apple, *Citrullus colocynthis*. Genet Resour Crop Ev. 54:327–336.

[CIT0008] Davis AR, Levi A, Tetteh A, Wehner T, Russo V, Pitrat M. 2007 Evaluation of watermelon and related species for resistance to race 1W powdery mildew. J Am Soc Hortic Sci. 132:790–795.

[CIT0009] Huseini HF, Darvishzadeh F, Heshmat R, Jafariazar Z, Raza M, Larijani B. 2009 The clinical investigation of *Citrullus colocynthis* (L.) Schrad fruit in treatment of type ii diabetic patients: a randomized, double blind, placebo-controlled clinical trial. Phytother Res. 23:1186–1189. 19170143 10.1002/ptr.2754

[CIT0010] Kousik CS, Shepard BM, Hassell R, Levi A, Simmons AM. 2007 Potential sources of resistance to broad mites (*Polyphagotarsonemus latus*) in watermelon germplasm. J Am Soc Hortic Sci. 42:1539–1544.

[CIT0011] Levi A, Thomas CE. 2005 Polymorphisms among chloroplast and mitochondrial genomes of *Citrullus* species and subspecies. Genet Resour Crop Ev. 52:609–617.

[CIT0012] Levi A, Thies JA, Ling KS, Harrison HF, Thomas CE, Simmons AM, Hassell R, Keinath AP. 2006 Novel watermelon breeding lines containing chloroplast and mitochondrial genomes derived from the desert species *Citrullus colocynthis*. Hortscience. 41:463–464.

[CIT0013] Levi A, Thies JA, Simmons AM, Harrison H, Hassell R, Keinath A. 2011 USVL-220, a Novel Watermelon Breeding Line. Hortscience. 46:135–138.

[CIT0014] Marzouk B, Marzouk Z, Haloui E, Fenina N, Bouraoui A, Aouni M. 2010 Screening of analgesic and anti-inflammatory activities of *Citrullus colocynthis* from southern Tunisia. J Ethnopharmacol. 128:15–19. 19962436 10.1016/j.jep.2009.11.027

[CIT0015] Robinson JT, Thorvaldsdottir H, Winckler W, Guttman M, Lander ES, Getz G, Mesirov JP. 2011 Integrative genomics viewer. Nat Biotechnol. 29:24–26. 21221095 10.1038/nbt.1754 PMC3346182

[CIT0016] Si Y, Zhang C, Meng S, Dane F. 2009 Gene expression changes in response to drought stress in *Citrullus colocynthis*. Plant Cell Rep. 28:997–1009. 19415285 10.1007/s00299-009-0703-5

[CIT0017] Tamura K, Stecher G, Peterson D, Filipski A, Kumar S. 2013 MEGA6: molecular evolutionary genetics analysis version 6.0. Mol Biol Evol. 30:2725–2729. 24132122 10.1093/molbev/mst197 PMC3840312

[CIT0018] Vashishta B. 2008 Antioxidant and free radical scavenging potential of *Citrullus colocynthis* (L.) Schrad. methanolic fruit extract. Acta Pharmaceut. 58:215. 10.2478/v10007-008-0008-1 18515231

[CIT0019] Zhu Q, Cui H, Zhao Y, Gao P, Liu S, Wang P, Luan F. 2016 The complete chloroplast genome sequence of the *Citrullus lanatus* L. Subsp. *Vulgaris* (Cucurbitaceae). Mitochondrial DNA Part B. 1:943–944. 10.1080/23802359.2016.1261611 PMC7800656 33473686

